# Retraction Note: Identification and functional characterization of key biomarkers in diffuse large B-cell lymphoma: emphasis on STYX as a prognostic marker and therapeutic target

**DOI:** 10.1186/s41065-026-00748-w

**Published:** 2026-10-09

**Authors:** Junaid Abid, Mahmood Basil A. Al-Rawi, Ahmad Mahmood, An Li, Tiemin Jiang

**Affiliations:** 1 State Key Laboratory of Food Nutrition and Safety, College of Biotechnology, Tianjin University of Science and Technology, Tianjin, 300222 China; 2 Department of Optometry, College of Applied Medical Sciences, King Saud University, Riyadh, 11433 Saudi Arabia; 3 Department of Hepatobiliary & Hydatid Diseases, Digestive & Vascular Surgery Center, The First Affiliated Hospital of Xinjiang Medical University, Urumqi, Xinjiang 830054 China; 4 State Key Laboratory of Pathogenesis, Prevention and Treatment of High Incidence Diseases in Central Asia, Xinjiang Medical University, Urumqi, Urumqi, 830054 China

**Retraction Note: Hereditas 162, 45 (2025)**

**https://doi.org/10.1186/s41065-025-00411-w**

The Editor-in-Chief has retracted this article. After publication, concerns were raised regarding the in vitro images presented in Fig. 9 of this article, where the patterns of cells and colonies do not appear consistent with natural cell growth and proliferation. It has also been noted that a number of references in this article do not appear to support the statements preceding the citations.

The Editor-in-Chief therefore no longer has confidence in the presented data.

An Li and Junaid Abid disagree with this retraction. The other authors have not responded to any correspondence regarding this retraction.

